# Sterilized Polyhexanide-Releasing Chitosan Membranes with Potential for Use in Antimicrobial Wound Dressings

**DOI:** 10.3390/membranes13110877

**Published:** 2023-11-08

**Authors:** Luís M. Vaz, Rita Branco, Paula V. Morais, António Jorge Guiomar

**Affiliations:** 1 Chemical Process Engineering and Forest Products Research Centre, Department of Life Sciences, University of Coimbra, Calçada Martim de Freitas, 3000-456 Coimbra, Portugal; luisvaz_9@hotmail.com; 2 Centre for Mechanical Engineering, Materials and Processes, Department of Life Sciences, University of Coimbra, Calçada Martim de Freitas, 3000-456 Coimbra, Portugal; rbranco@uc.pt (R.B.); pvmorais@uc.pt (P.V.M.)

**Keywords:** polyhexanide, poly(hexamethylene biguanide), polyhexamethylene biguanide, PHMB, chitosan, membrane, controlled drug release, drug delivery, wound dressing, antimicrobial

## Abstract

Wound infection is a common complication of chronic wounds. It can impair healing, which may not occur without external help. Antimicrobial dressings (AMDs) are a type of external help to infected chronic wounds. In this study, highly porous membranes made of only chitosan and containing the antiseptic polyhexanide (poly(hexamethylene biguanide); PHMB) were prepared by cryogelation, aiming to be used in AMDs. These membranes exhibited a water swelling capacity of 748%, a water drop penetration time of 11 s in a dry membrane and a water vapor transmission rate of 34,400 g H_2_O/m^2^/24 h when in contact with water. The best drug loading method involved simultaneous loading by soaking in a PHMB solution and sterilization by autoclaving, resulting in sterilized, drug-loaded membranes. When these membranes and a commercial PHMB-releasing AMD were assayed under the same conditions, albeit far from the in vivo conditions, their drug release kinetics were comparable, releasing PHMB for ca. 6 and 4 h, respectively. These membranes exhibited high antibacterial activity against *Escherichia coli*, *Staphylococcus aureus* and *Pseudomonas aeruginosa*, which are bacterial species commonly found in infected wounds and blood clotting activity. The obtained results suggest that these membranes may have potential for use in the development of AMDs.

## 1. Introduction

Wounds are a disruption of the normal anatomical structure and function [1] due to physical, chemical or thermal injury. In open wounds, the disruption of skin continuity provides a surface with moisture, temperature and nutritional conditions suitable for microbial proliferation and colonization, often resulting in infection. The occurrence of infection compromises the natural wound healing process, a sequence of events through which the skin recovers its anatomical and physiological integrity [2,3]. When it fails to achieve a persistent structural and functional restoration within a 5 to 10 days timeframe (although it can take as long as 30 days), the wound is termed chronic [1,4]. Chronic, non-healing wounds have a huge impact on the health and quality of life of patients and their families. Wound care poses a substantial financial burden to the healthcare system and the increase in population with an advanced age, together with the increase in the incidence of diabetes and obesity worldwide, are contributing to the rising costs of wound care [5].

Chronic wound healing is unlikely to be successful without external aid. This failure may be due to repeated injury, existence of pathological conditions (such as poor primary treatment, malignancies, diabetes and other pathologies) or to other factors, such as persistent infection, hypoxia, necrosis and excessive levels of inflammatory cytokines and of exudate, the liquid medium present in the wound. If left untreated, it can result in serious complications, such as limb amputation, sepsis and even death [5]. Currently, procedures that lead to rapid and effective healing of wounds are still lacking. The most common external aid to the wound healing process is the use of wound dressings (WDs). In their simplest form (passive WDs), they are matrices made of one or more natural, modified natural or synthetic polymers, most commonly in the form of a sheet or pad. They have the role of a selective barrier, preventing the entrance of microorganisms and other foreign agents present in the outside environment, while allowing the transport of water vapor, oxygen and carbon dioxide to or from the outside environment, as well as absorbing excess exudate. Bioactive or medicated WDs are another type of WD. They actively help the wound healing process by combating infection and/or promoting wound healing. As infection occurs often in chronic wounds, increasing the risk of life-threatening complications, a variety of WDs that contain antimicrobial agents (antimicrobial dressings or AMDs) have been developed and are commercially available. Yet, despite the wide variety of commercially available WDs, the number of AMDs that have reached the market is still limited, due to high production costs, poor drug stability, challenging storage conditions and difficulties in creating drug-loaded WDs that preserve the full therapeutic effect of the drug [6,7]. In addition, wound care professionals consider that none of the commercially available AMDs show optimal and broad antimicrobial power [8]. Thus, more research in AMDs is needed.

**Figure 1:**
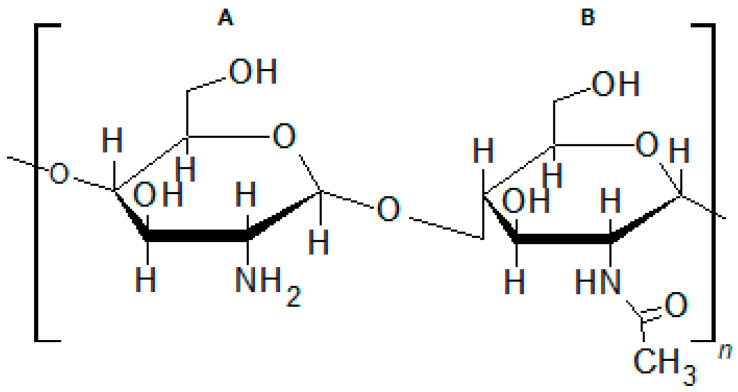
Chemical structure of commercial chitosan. (**A**) D-glucosamine; (**B**) *N*-acetyl-D-glucosamine.

Commercial WDs are made from a variety of polymers [9]. Chitosan (CS) is a biopolymer that has attracted great attention for use in WDs [10] and in controlled drug delivery [11]. In fact, research has already produced a number of commercially available CS-based WDs [10,12]. Chitosan is commonly obtained via alkaline deacetylation of chitin, the second most abundant polysaccharide on Earth [13]. It is a sustainable biopolymer, since chitin is obtained mainly from crustacean shells, an abundant waste of the food industry [14]. In its commercial form, it is not fully deacetylated, being a linear polysaccharide composed of randomly distributed D-glucosamine units linked to *N*-acetyl-D-glucosamine through β(1→4) glycosidic bonds (Figure 1). CS has antimicrobial and blood clotting activity, stimulates wound healing and is nontoxic and biocompatible [15,16,17,18]. These properties make it suitable for use in WDs. Being soluble in acidic aqueous solutions, but not soluble in alkaline solutions, in ethanol and other organic solvents, preparation of CS membranes is possible via a wide variety of methods [10,19]. In this study, porous CS cryogels in the form of membranes were prepared by cryogelation [20], a method that allows the production of porous, membrane-type cryogels made of neat CS. These membranes were loaded with an antimicrobial agent by soaking in the antimicrobial agent solution, aiming to be used in AMDs. As WDs have to be sterile, the antiseptic-loaded membranes were also sterilized.

**Figure 2:**
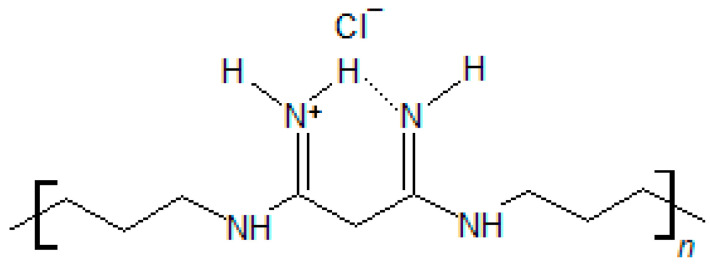
Chemical structure of the repeating unit of the hydrochloric salt of PHMB.

The antimicrobial agent selected for this study was polyhexanide—poly(hexamethylene biguanide), abbreviated to PHMB—mostly used as its hydrochloric salt. It is a synthetic polydisperse mixture of positively charged oligomers, composed of alternating biguanide and hexamethylene sequences (Figure 2), that has been widely employed as an antiseptic since the 1950s [21]. In addition to its use in wound antisepsis, where it has been considered the first choice for burns and for critically colonized and infected chronic wounds, as well as one of two first choices for the treatment of contaminated acute and chronic wounds [22], PHMB has found a wide range of other applications. It has been used as an antiseptic in a variety of both medical and non-medical settings [23,24,25] and in other applications as diverse as gene delivery [26], improvement of cotton fabric dyeability [27] or CO capture and sensing [28]. As an antimicrobial agent, PHMB is a potent antiseptic, with minimum inhibitory concentrations (MIC) and minimum biocidal concentrations (MBC) in the low µg/mL range [29]. It is effective against a wide range of both Gram-positive and Gram-negative bacteria (including difficult to control bacterial strains) [30], yeasts and other fungi [31], amoeboids [32] and some enveloped [33,34] and non-enveloped viruses [35]. When applied to wounds, PHMB is well tolerated, has low cytotoxicity [31] and stimulates wound healing [36]. Its mechanism of action relies on (i) binding to the negatively charged cell membranes and walls, resulting in cell lysis due to disruption of membrane integrity [21,37], (ii) binding to negatively charged phospholipids in the cell membrane, causing impairment of ion pumps, receptors and cell membrane enzymes [21,37], and (iii) binding and condensing bacterial chromosomes, arresting cell division. PHMB shows specificity for bacterial cells since eukaryotic cells have a relatively neutral surface [38]. Although PHMB can enter eukaryotic cells, it does not damage the cell membrane and does not enter the nucleus, being trapped within endosomes, which are absent in bacteria [38,39]. Furthermore, its non-specific mechanism of action makes it unlikely that bacteria will develop resistance to PHMB. In fact, there have been no reports of resistance to PHMB in its normal use, even though it has been in use for eight decades [40]. Given these characteristics, together with its good thermal and hydrolytic stability [41,42], as well as its solubility in water [23], PHMB is well suited to be included in formulations to develop AMDs. In fact, it is already present in several commercial AMDs [43]. Nevertheless, since it is an oligomer, its molecular dimensions are larger than those of typical drug molecules. Additionally, in water, it may form polymeric micelles of the core/shell type, in which its hydrophobic segments point towards the center of a sphere (core), while its hydrophilic groups point outwards (shell) [44]. At concentrations normally employed in wound antisepsis and in commercial PHMB-releasing AMDs (0.01%–0.05% [22]), micelle formation is not expected, as PHMB’s critical micellar concentration (CMC) is considerably higher (0.02–0.05 M in water [44], equivalent to ca. 50–130 mg/mL or 5–13%). On the other hand, in water and at concentrations below its CMC, PHMB may form large aggregates with particle dimensions in the micrometer range [44]. This may make it more challenging to load PHMB into polymeric matrices by soaking, as well as its release, in comparison to the typical drugs used.

Although a few CS-based hydrogels that release PHMB aimed to be used in AMDs have been reported in the literature [45,46,47,48,49,50,51,52,53,54], PHMB-releasing, CS-based AMDs are not yet commercially available [43]. In this work, a contribution to the development of antimicrobial PHMB-based membranes made of CS, aimed at an application as AMDs, is presented. Cryogels in membrane form made of CS only, i.e., without the use of other substances, such as plasticizers or crosslinkers, were produced by cryogelation and loaded with PHMB. This is beneficial as it eliminates the possibility of biocompatibility issues or PHMB quantification errors caused by leaching of additional reagents. Comparable CS-based, PHMB-releasing membranes for AMDs required other polymers in addition to CS, such as PEO [45], alginate [46] or PVA [50], crosslinkers [45,54] or surfactants [46].

Some physicochemical characteristics of the resulting membranes, such as morphology, swelling capacity, wettability, water penetration, moisture vapor transmission and the PHMB release kinetics were evaluated, as well as their antimicrobial activity and in vitro blood clotting activity. Care was taken to address the relevance of the obtained drug release results in view of the intended application. As such, the release studies were carried out with sterilized drug-loaded membranes. This has not been the case with PHMB-releasing membranes [43] and is scarcely undertaken in studies that employ other drugs. The study of sterilized samples is necessary, since WDs have to be sterile and sterilization can alter the characteristics of polymers and drugs [55], as well as the drug release kinetics [56]. In addition, published drug release assays with membranes that release antibacterial agents for application as WDs have been carried out in a variety of conditions, all very far from the conditions found in the wound bed [43]. As such, the drug release duration and the cumulative drug release levels obtained in studies carried out employing those models of the wound bed are not relevant. In the drug release studies carried out in this work, the following approaches were adopted, in order to improve the relevance of the model and of the obtained results: (i) drug release from a single face of the membrane was studied, as a WD will contact the wound bed through a single side only; (ii) the use of a release medium with relevant pH and temperature, as well as an ionic strength comparable to that of the wound exudate; and (iii) in an attempt to infer about the in vivo relevance of the drug release profiles obtained, although without avoiding assay conditions far from the in vivo conditions (in particular, the volume, composition, viscosity and turnover rate of the release medium, as well as the use of samples fully immersed in the release medium), the drug release profiles of the prepared membranes and of a commercial wound dressing that releases PHMB were assayed under the same conditions and compared. Similar drug release profiles may indicate that the test membranes exhibit an in vivo drug release behavior similar to that of commercial WDs, and therefore, may be appropriate for an application as WDs.

## 2. Materials and Methods

### 2.1. Preparation of Chitosan Membranes

CS membranes were prepared by employing a cryogelation method inspired by the method reported by Hsieh et al. [20]. It consisted of the following steps: (i) preparation of an aqueous 3 wt% CS solution (CS deacetylation percentage: ≥75%; Acros Organics, Belgium) in 0.2 M acetic acid (≥99.8%; Thermo Fisher Scientific, Waltham, MA, USA); (ii) transfer of 3 g of this CS solution to a mold consisting of a glass Petri dish with an internal diameter of 5 cm; (iii) freezing this CS solution in the mold at −20 °C for 8 h; and (iv) coagulation of the frozen CS solution at −20 °C by immersion of the mold in a 1 M NaOH (≥99%; Carl Roth GmbH & Co., Karlsruhe, Germany) solution in 25:75 ethanol/water (ethanol: 96%; JMGS, Odivelas, Portugal) contained in a beaker also at −20 °C for 16 h. After removal from the coagulation solution and thawing, the formed membranes were lifted off the mold with the aid of a spatula and washed in a controlled manner. The washing process involved suspending the membranes in distilled water and in PBS (Phosphate Buffered Saline, 0.14 M NaCl, 2.7 mM KCl, 10 mM phosphate, pH 7.4) under magnetic stirring, with periodic renewal of the washing solutions until the ultraviolet (UV) spectrum of the washing solution between 200 and 300 nm, and especially at 236 nm (wavelength at which PHMB absorbs to the maximum), was residual. Discs with the required diameter were then cut with a cork borer in zones of the membrane away from the edge and stored at 4 °C in sealed plastic pouches, to prevent dehydration.

### 2.2. PHMB Loading

Discs with a diameter of 1.9 cm were placed in 15 mL Falcon tubes containing 5 mL of PHMB solutions (PHMB hydrochloride, ≥94%; Biosynth Carbosynth, Compton, UK) with concentrations of 0.1 mg/mL, 1 mg/mL, 2 mg/mL and 5 mg/mL in PBS. These tubes were incubated for 24 h or 72 h at 34 °C in a shaking incubator (shaking speed: 100 rpm). The drug loading was carried out in triplicate (1 disc per tube). A sample of the PHMB loading solution employed in this loading step was also incubated under the same conditions, so that it would be possible to account for any incubation effects in the PHMB solution.

In a second drug loading method, the discs were loaded with PHMB during sterilization by autoclaving. Discs with a diameter of 1.9 cm placed in 15 mL Falcon tubes (1 per tube, in triplicate) containing 5 mL of a 5 mg/mL PHMB solution in PBS were autoclaved in a Prestige Medical autoclave, model Omega Media (Prestige Medical, Blackburn, UK). Samples of the PHMB solution used for drug loading were also autoclaved in parallel, so that it would be possible to account for any incubation effects in the PHMB solution. The autoclaving cycle was carried out at 121 °C and 1.4 atm for 22 min. Care was taken so that the time spent inside the autoclave after the end of the autoclaving cycle was always the same whenever samples were autoclaved. In a variation of this drug loading/sterilization method, the discs remained in the PHMB solution for 24 h at 34 °C after autoclaving.

### 2.3. PHMB Quantification

The concentration of PHMB in solution was determined by UV absorption spectroscopy, through measurement of the absorbance at 236 nm in a VWR UV-6300PC spectrophotometer (VWR, Radnor, PA, USA). A calibration curve was obtained employing PHMB solutions of known concentration in the range of 1 to 43 µg/mL of PBS, in triplicate.

The quantification of the PHMB loaded into the membranes was calculated by mass balance, measuring the absorbance at 236 nm of the drug loading solution after the loading step (in triplicate) and that of a sample of the PHMB solution used for loading that was incubated under the same conditions of the discs, but in the absence of discs (in triplicate). The absorbance was converted to concentration employing the mentioned calibration curve. The amount of PHMB loaded was expressed as mass of PHMB loaded per mass of dry membrane (µg PHMB/mg of dry membrane).

### 2.4. Physicochemical Characterization of the Prepared Membranes

#### 2.4.1. Morphology of Cross-Sections

Samples that were dried at 40 °C were immersed in liquid nitrogen, fractured manually and mounted on the microscope stubs so that the fracture surface was visible. They were then coated by magnetron sputtering with a 10–15 nm thick layer of gold, using an Edwards EDX Sputter Coater (Edwards Vacuum, Burgess Hill, UK). Finally, the samples were observed with a Zeiss Merlin Compact VP FE-SEM electron microscope (Carl Zeiss AG, Jena, Germany), using an accelerating voltage of 2 kV.

#### 2.4.2. Membrane Thickness and Swelling Capacity

The thickness of the dry and hydrated membranes was measured employing a caliper with a resolution of 0.02 mm. It was measured in 3 different positions of 3 membranes.

To determine the swelling capacity, discs with a diameter of 1.9 cm were placed in an oven at 40 °C until a constant dry mass was obtained. Subsequently, the dry discs were immersed in PBS at room temperature until a constant hydrated mass was obtained. The swelling capacity was expressed as the percent water absorbed in relation to the mass of the dry disc. This assay was carried out in triplicate.

#### 2.4.3. Contact Angle Goniometry and Drop Penetration Rate

The measurement of contact angles was performed using the sessile drop method, employing a KSV-CAM 101 goniometer (KSV Instruments Ltd., Helsinki, Finland). A droplet of Milli-Q water with a volume of approximately 4 µL was deposited on the surface using the goniometer’s microsyringe. Images of the drop’s profile were captured with the goniometer’s video camera and, with the help of the goniometer’s software (Attension Theta, v. 4.1.0, KSV Instruments Ltd., Helsinki, Finland), the contact angle of each image was automatically determined by fitting of the Young–Laplace equation to the drop’s profile. The capture of the drop’s images was carried out automatically after a fixed drop residence time on the surface of 1 s. Images were then captured with an interval of 0.10 s for 60 s and a plot of contact angle versus time was obtained. The contact angle value of each drop was the average of the contact angles on the left and right sides of the drop in the region of the plot where the contact angle remained constant. Measurements were carried out in 3 replicate membranes in 3 different locations on their surface. Both dry samples and samples hydrated in PBS were studied. In the latter case, after reaching swelling equilibrium in PBS, the surface of the samples was blotted with absorbent paper immediately before the determination. To avoid membrane dehydration, this procedure was repeated before each individual contact angle measurement.

The penetration rate of a water droplet, as well as the penetration time, were determined by using the video camera of the contact angle goniometer to track the movement of a water droplet as it penetrated the sample. For this, a 4 µL droplet was placed on the sample surface and the time it took to fully penetrate the sample surface was determined from the video captured. The rate of penetration was determined from a plot of the height of the drop above the membrane surface versus time. For this, a linear regression was carried out with the data points in the steady-state phase of drop penetration (the linear portion of the curve), taking the absolute value of the slope of this straight line as the drop penetration rate. Both dry membranes and membranes pre-hydrated in PBS and thoroughly blotted with absorbing tissue paper were evaluated. For each type of sample, measurements were performed in 3 different locations, and between 4 and 5 replicate samples were studied.

#### 2.4.4. Moisture Vapor Transmission Rate

The moisture vapor transmission rate (MVTR) was determined according to the EN 13726-2:2002 standard [57], employing the two different methods mentioned in this standard: (i) assay with sample in contact with water vapor and (ii) assay with sample in contact with water. Each of these assays was performed with 6 membranes and was repeated. Before the assays, dry sample discs of 0.8 cm in diameter were conditioned inside a desiccator at a relative humidity (RH) of ca. 75% and at room temperature, until reaching a constant mass.

The sample in contact with water vapor assay was carried according to the indications of Section 3.2 of the EN 13726-2:2009 standard [57], albeit employing (i) a different cell type, (ii) a sample with a different area and (iii) a different water volume. This assay is a gravimetric assay in which permeability cells (Paddington cups) with an opening with an area of 10 cm^2^, where the sample is mounted, are employed. In this study, the Paddington cups were replaced by cylindrical glass flasks whose opening was fitted with the samples, fixed in place with lids that possessed a circular opening in the center with an area of 0.26 cm^2^. The flasks contained 1.75 mL of Milli-Q water, a volume that allowed for the 5 mm spacing between the surface of the water and the sample required by the standard. The flasks with water and the samples were weighed (*m*_1_) and placed upright in a desiccator whose interior was at 37 °C and at a RH of less than 20% (achieved using silica gel), which also contained a small fan. After 24 h, the flasks with the samples were weighed again (*m*_2_) and the MVTR was calculated from the mass of water lost as vapor (*m*_1_–*m*_2_) per square meter per 24 h.

For the sample in contact with water assay, Section 3.3 of the EN 13726-2:2009 standard [57] was followed, with the same deviations already mentioned for the water vapor in contact assay. The procedure of both assays was similar, with the exception that, in the water-in-contact assay, the flasks containing water and fitted with the samples were inverted and placed in a holder that ensured that there was a gap under the membrane to allow airflow. Care was taken to ensure that the flasks were not leaking.

#### 2.4.5. Thermogravimetric Analysis

The samples to be analyzed by Thermogravimetric Analysis (TGA) were dried in an oven at 40 °C and kept in a desiccator with silica gel until they were analyzed. The TGA thermogram was obtained employing a TGA Q500 analyzer (TA Instruments, New Castle DE, USA), with a heating program running from 25 °C to 600 °C, at a constant heating rate of 10 °C/min, in a nitrogen atmosphere. In addition to the thermogram, the Differential Thermogravimetry (DTG) curve was obtained from the first derivative of the TGA thermogram.

#### 2.4.6. Fourier Transform Infrared Spectroscopy

Fourier transform infrared (FTIR) spectra were obtained in a Jasco FTIR 4200 spectrometer (Jasco, Tokyo, Japan), equipped with an ATR Golden Gate MKII accessory (Specac, Orpington, UK), which had a diamond crystal with an angle of incidence of 45°. The samples to be analyzed were dried in an oven at 40 °C and kept in a desiccator with silica gel until they were analyzed. The analysis was carried out with a spectral resolution of 2 cm^−1^ and an accumulation of 64 spectra. The baseline obtained was corrected and the spectra were normalized by dividing each absorbance by the maximum value of absorbance observed in each spectrum.

### 2.5. PHMB Release

#### 2.5.1. PHMB Release from a Single Face

**Figure 3:**
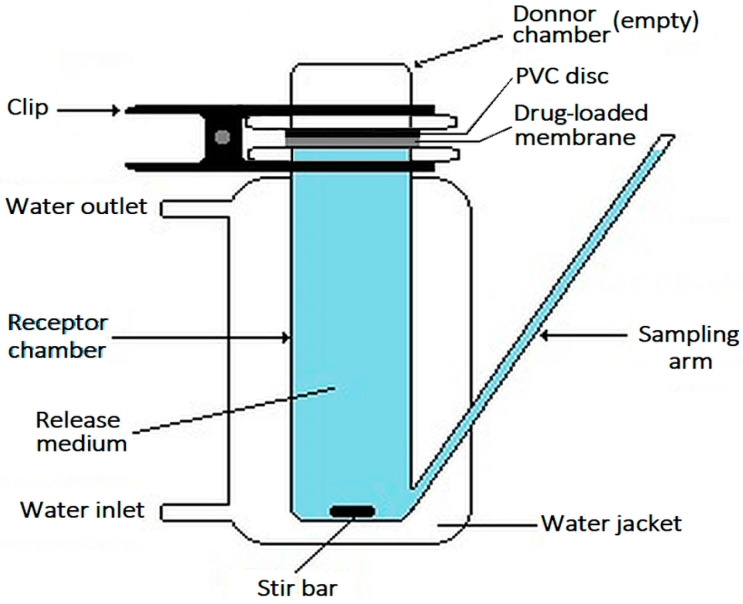
Schematic representation of the Franz diffusion cell as employed in the drug release studies.

Calibrated Franz diffusion cells [58] (Soham Scientific, Ely, UK) with a capacity of 14.55 mL and an opening with an area of 1.77 cm^2^ were employed to study the PHMB release kinetics from a single face of the membranes. PHMB-loaded discs of 1.9 cm in diameter, that were loaded and not allowed to dry before use, were employed. After blotting with absorbent paper tissue, the discs were placed between the two cell chambers (Figure 3). The donor chamber was empty, while the receptor chamber contained PBS (pH 7.4) at 34 °C and was magnetically stirred at ca. 330 rpm. A transparent, impermeable PVC disc was placed in contact with the upper face of the membranes to be studied to prevent loss of PBS through the membrane. At different time intervals, 400 µL aliquots were collected from the receptor chamber and immediately replaced with an equal volume of PBS. After diluting the aliquots with PBS, their absorbance at 236 nm was measured and converted to PHMB concentration (Section 2.3). The drug release curve was obtained through plotting the cumulative mass of drug released per unit mass of dry membrane as a function of time. The conditions of this assay provided infinite sink conditions throughout the assay (confirmed by determining the PHMB concentration in the solution at each time). To take into account the absorbance of some CS or AA that could be released by the membranes and absorb at the same wavelength employed in PHMB quantification, release assays were also performed with CS membranes not loaded with PHMB but submitted to the same loading conditions as the membranes loaded with PHMB, however, in a PBS solution that did not contain PHMB (blanks). These assays were carried out in triplicate, employing three Franz diffusion cells simultaneously.

#### 2.5.2. PHMB Release in Batch

Squares with sides of 1.5 cm were cut from a commercial AMD that releases PHMB (Telfa^®^ AMD^TM^; Cardinal Health, Dublin, OH, USA), with the resulting open edges sealed using a thermal sealer (Impulse Sealer Type F-250; Vimi, Hangzhou, China), so that no drug would be released through the open edges. These samples as well as dry discs of 1.9 cm in diameter of PHMB-loaded membranes were assayed in batch in a low volume, i.e., with the samples fully immersed in 2.0 mL of PBS (pH 7.4) at 34 °C, under magnetic stirring at approximately 500 rpm. Aliquots of 400 µL were periodically collected and immediately replaced by an equal volume of PBS. The assay was carried out in triplicate and the drug release curve was obtained as in Section 2.5.1.

### 2.6. Biological Characterization

#### 2.6.1. Antibacterial Activity

The antibacterial activity of the PHMB solution was evaluated through the determination of its MIC against 3 bacterial strains, employing the broth dilution method. The strains employed were *Escherichia coli* (ATCC 25922), *Staphylococcus aureus* (ATCC 25923) and *Pseudomonas aeruginosa* (DSM 1117), belonging to the University of Coimbra Bacteria Culture Collection (UCBCC). Luria-Bertani (LB) liquid medium was prepared by dissolving 10 g of tryptone, 5 g of yeast extract and 5 g of sodium chloride in 1 L of deionized water. Stock solutions of 5 mg/mL PHMB were prepared in PBS (pH = 7.4) and sterilized by two different methods: (i) membrane filtration through a 0.2 µm pore size membrane or (ii) autoclaving for 20 min at 121 °C and 1.4 atm. The assays were performed employing serial 2-fold dilutions of PHMB solutions prepared in LB growth medium using 96-well microliter plates. The range of PHMB concentrations in the wells was 0.07–100 µg/mL for *E. coli* and *S. aureus* strains and 0.14–200 µg/mL for *P. aeruginosa*. Each well containing 150 μL of PHMB solutions was inoculated with 50 μL of bacterial suspensions prepared in LB medium (10^6^ CFU mL^−1^) and further incubated at 37 °C for 24 h. Subsequently, 1 mL of a bacterial suspension with a cell density equivalent to the 0.5 McFarland standard (1.5 × 10^8^ CFU/mL) was added to each well. Positive controls (medium without PHMB) were performed for each bacterial strain. Additionally, negative controls (LB medium containing PHMB but no added bacterial suspension) were also prepared to check the natural turbidity of the tested PHMB solutions. After incubation, bacterial growth was evaluated by measuring the optical density at 600 nm (OD_600_) with the aid of a microplate reader (Tecan Infinite 200 PRO, Tecan, Männedorf, Switzerland). The MIC was taken as the lowest concentration of PHMB that completely inhibited bacterial growth. Each assay was carried out in duplicate for each bacterial suspension.

The antibacterial activity of the membrane samples was determined in two different ways: (i) in bacterial suspensions and (ii) in bacterial cultures in agar plates. In the first method, bacterial suspensions of a Gram-positive (*S. aureus*) and of a Gram-negative (*P. aeruginosa*) bacteria, having an optical density at 600 nm equal to 0.06 (measured in a Thermo Scientific Evolution 201/220 UV-Visible Spectrophotometer, Thermo Fisher Scientific, Waltham, MA, USA), were prepared by dilution of the starting suspension with LB medium. A volume of 1.5 mL of each bacterial suspension was placed in the wells of a 24-multiwell plate containing the disc to be assayed. Each disc had a diameter of 1 cm and was blotted with sterile absorbent paper before being placed in the well. This assay was carried out in duplicate for discs loaded/autoclaved in a 5 mg/mL PHMB solution in PBS and for control discs (discs autoclaved in PBS without PHMB). The multiwell plate was incubated in a shaker at 37 °C, under agitation at 130 rpm. Periodically, 100 µL aliquots were collected and their OD_600_ was measured employing a Tecan Infinite 200 PRO absorbance microplate reader to obtain the bacterial growth curve.

In the second method, a sample of 100 µL of the tested bacterial suspensions, with a cell density equivalent to the 0.5 McFarland standard, was spread on Mueller–Hinton (MH) agar plates. Then, sample discs loaded/autoclaved in a 5 mg/mL PHMB solution in PBS with a diameter of 1 cm were blotted with sterile absorbent paper and placed directly on the inoculated plates that were then incubated for 24 h at 37 °C, at conditions of high humidity to prevent dryness. After incubation, the plates were observed and the diameters of the inhibition halos were measured. Control discs autoclaved in PBS without PHMB were also tested. Each assay was performed in duplicate for each bacterial suspension.

#### 2.6.2. Blood Clotting Activity

To assess whether the drug-loaded CS membranes can cause blood to clot, the thrombosis degree induced by the membranes was determined employing a colorimetric variation of Imai and Nosé’s in vitro gravimetric assessment of the thrombogenicity of materials [59]. In this variation [60], the thrombosis degree was obtained from the hemolysis degree of blood that contacted the sample, through a measurement of the absorbance at 545 nm. Strips of 2.1 × 0.5 cm were cut from the CS membranes and loaded/autoclaved in a 5 mg/mL PHMB solution, under the same conditions mentioned in Section 2.2, employing the same (sample area)/(PHMB solution volume) ratio. The samples (in quadruplicate, assayed individually) were blotted with an absorbent tissue, immersed in 1 mL of pooled fresh rabbit blood anticoagulated with ACD-A (Acid Citrate Dextrose Solution A), supplied by Probiológica (Belas, Portugal), contained in plastic 1.5 mL microcentrifuge tubes and incubated at 37 °C. The assay was started by recalcification with 150 µL of a 25 mM CaCl_2_ aqueous solution (Carl Roth Gmbh & Co., Karlsruhe, Germany). After 45 min, 1 mL of Milli-Q water was added to each tube to cause hemolysis of the blood cells not trapped in the blood clot, incubated at 37 °C for 5 min and centrifuged for 5 min at 3000× *g* (Eppendorf Minispin centrifuge, Eppendorf SE, Hamburg, Germany). Subsequently, a sample of the supernatant was collected and diluted with PBS in order to obtain an absorbance below 1 at 545 nm, employing a Thermo Scientific Genesys 20 Visible Spectrophotometer (Thermo Fisher Scientific, Waltham, MA, USA). The following controls, also in quadruplicate, were assayed in parallel with the samples: (i) a tube containing the same volumes of blood and CaCl_2_ but containing PBS instead of water and not containing a sample strip, that will allow to account for any hemoglobin already present in the plasma at the start of the assay; (ii) a tube containing the same volumes of blood, CaCl_2_ solution and water, but not containing a sample strip, in which full hemolysis of blood will occur. The thrombosis degree was obtained employing Equations (1) and (2), where *A*_545_(sample) is the absorbance at 545 nm obtained with the samples, *A*_545_(plasmaHB) is the absorbance at 545 nm obtained with the starting non-hemolyzed blood and *A*_545_(totalHb) is the absorbance at 545 nm obtained with the fully hemolyzed blood.

Thrombosis degree (%) = 100% − Hemolysis degree (%)(1)

Hemolysis degree (%) = 100% × [*A*_545_(sample) – *A*_545_(plasmaHb)]/[*A*_545_(totalHb) – *A*_545_(plasmaHb)] (2)

### 2.7. Statistical Analysis

Comparisons between the means of two groups employed a two-tailed, unpaired Student’s *t* test. For comparisons between more than two groups, one-way ANOVA was employed, followed by Tukey’s HSD test. All statistical analyses were conducted at a 95% confidence level. The standard deviation (SD) was used as a measure of spread in the data.

## 3. Results and Discussion

This study aimed to produce CS membranes that release the antiseptic PHMB, for use in AMDs. Given the large molecular size of PHMB and the possible formation of molecular aggregates [44], and as PHMB will be loaded into the membranes by soaking the membranes in PHMB solutions, a membrane preparation method that could produce highly porous membranes was selected. In the selected method, a polymer solution was frozen and subsequently coagulated in a non-solvent at a temperature below the melting point of the frozen polymer solution [20]. During the freezing step, phase separation occurs, forming a polymer-rich phase and an ice-rich phase; upon melting, the ice-rich phase will become the porous phase. The CS-rich phase was coagulated by immersing the frozen CS solution in a non-solvent solution, an alkaline solution containing ethanol. To prevent the frozen polymer solution from melting and losing its porous structure, the coagulating solution was at the same temperature at which the polymer solution was frozen. In this step, an increase in the non-covalent intermolecular and intramolecular interactions between the CS polymeric chains occurs, such as hydrophobic interaction and hydrogen bonding, resulting in a more compact and insoluble porous CS network. The pore size and density can be altered by adjusting process variables that determine the characteristics of the ice phase, such as the freezing temperature and the freezing rate, among others [20]. This method allows for the preparation of highly porous membranes made of only CS, which eliminates the need for crosslinkers or plasticizers. This has the advantage of excluding the risk of biocompatibility complications or PHMB quantification errors caused by leaching of additional reagents. To ensure sterility, the PHMB-loaded membranes were sterilized by autoclaving.

### 3.1. Physicochemical Characterization

#### 3.1.1. Morphology

**Figure 4:**
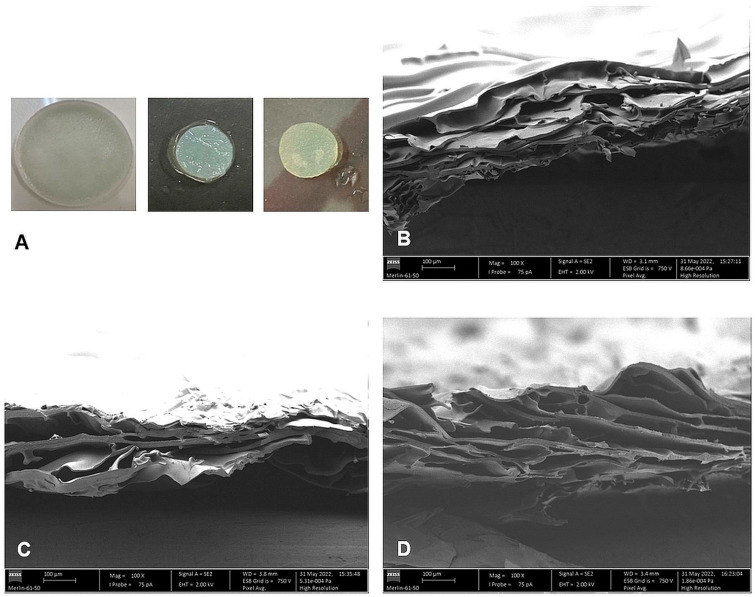
Photographs and SEM micrographs (magnification: 100×) of the CS membranes. (**A**) Photographs of a CS membrane after being removed from the mold (left) and of wet, swollen discs cut from the CS membrane after washing (center) and after autoclaving (right); (**B**–**D**) SEM micrographs of cross-sections of CS membranes without PHMB (**B**), loaded with PHMB by soaking for 24 h at 34 °C (**C**) and loaded with PHMB by simultaneous soaking and sterilization by autoclaving (**D**).

The obtained membranes were pale white, with a uniform and smooth appearance (Figure 4A). They did not dissolve in PBS. When hydrated, they were flexible, could be bent without fracturing and exhibited some resistance to pulling by hand. Their thickness was ca. 220 µm when dry and ca. 1200 µm when hydrated (Table 1) and their swelling capacity (748%; Table 1) was within the range of the swelling capacity of a set of commercial WDs reported in the literature [61].

The internal morphology of the dry CS membranes was analyzed employing Scanning Electron Microscopy (SEM) to observe cross-sections of the samples. The cross-section of the membranes had a stratified porous appearance (Figure 4B), with large, elongated pores in the micrometer range, confirming that the method selected produced porous membranes. Although PHMB can form micrometer-size aggregates in water [44], no such aggregates were observed on the membranes loaded with PHMB (Figure 4C), even at higher magnification. It is likely that these aggregates that can form in water did not form in the PBS solution employed in drug loading. In effect, molecular dynamics simulation studies have suggested that, in aqueous solutions that contain monovalent counterions, PHMB can self-assemble into compact, ordered hairpin-like structures of much lower dimensions than the mentioned aggregates, with radii of gyration of ca. 1–2 nm [62,63]. They are formed in a process not mediated by ions or water molecules but by the counterintuitive interaction of like-charge biguanidinium ions [62,64].

#### 3.1.2. Wettability

The wettability of the membranes was evaluated using contact angle goniometry. The water contact angle for dry and for hydrated CS membranes was 97 ± 2° and 82 ± 6°, respectively (Table 1). According to the Berg limit, which sets the dividing line between hydrophilic and hydrophobic surfaces at 65° [65], both the dry and hydrated surfaces can be classified as hydrophobic but, when using the traditional dividing line value of 90°, the surface of the dry membranes is classified as hydrophobic, while the hydrated surface is classified as hydrophilic, albeit close to this hydrophilic/hydrophobic dividing line. The water contact angles of the dry CS membranes were comparable to those reported in the literature for membranes made of only CS, although prepared by different methods (e.g., 96° [66]). However, our results could not be compared to results obtained with commercial WDs, as no contact angle values for commercial WDs made only of CS were found in the literature.

#### 3.1.3. Water Penetration

**Figure 5:**
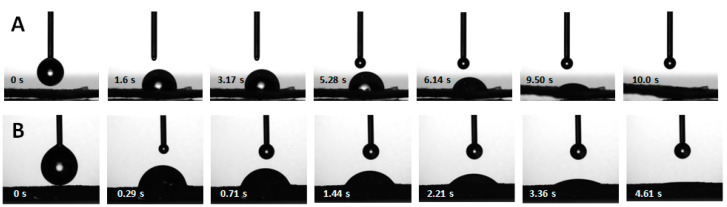
Selected sequence of images representing the penetration of water drops into dry (**A**) and pre-hydrated, non-saturated (**B**) membranes.

Although the surfaces of the CS membranes were not explicitly hydrophilic, the membranes absorbed water, showing a high water swelling capacity (Table 1). As WDs must be able to quickly absorb the exudate produced by the wound, a study of the penetration of water droplets on the membrane’s surfaces was undertaken. Water droplets placed on the membranes quickly penetrated the surface (Figure 5). Water penetration in a hydrophobic porous surface can occur when the droplets are in the Wenzel state, in which they fully wet a rough surface, as opposed to the Cassie–Baxter state, in which they are supported by air trapped in the topography of a rough surface and do not penetrate it [67,68,69]. To characterize the water penetration kinetics, the penetration time of droplets of water placed on the membranes’ surface was measured and the penetration rate was calculated. In addition to dry membranes, pre-hydrated, non-saturated membranes were also studied, to determine if membranes that had absorbed some water could still quickly absorb more water. In Figure 5, a sequence of images illustrating the penetration of a water droplet in dry and pre-hydrated, non-saturated membranes is presented. After just over 10 s, the drop fully penetrated a dry membrane (Figure 5A), with an average penetration time of 11 ± 4 s (Table 1). When the membranes had already absorbed some water, the water droplets penetrated the surface almost three times faster (Figure 5B). The average penetration time for the pre-hydrated membranes was 4.1 ± 0.6 s (Table 1). This indicates that membranes that have already absorbed water can quickly absorb more water. As the penetration of water droplets in dry membranes occurs concomitantly with the expansion of the membrane due to its hydration, this could explain the faster penetration times in pre-hydrated membranes.

For dry membranes, drop penetration exhibited two linear phases: a slow first phase, at the beginning of the process, with a penetration rate of 0.02 ± 0.01 mm/s, followed by a second faster phase, with a penetration rate of 0.10 ± 0.02 mm/s (Table 1). As expected, given the drop penetration times obtained, the penetration rate in dry membranes was lower than in pre-hydrated, non-saturated membranes. For the latter, a single linear phase was obtained, with an average penetration rate of 0.24 ± 0.05 mm/s (Table 1). The occurrence of two water penetration phases in the case of dry membranes may be due to the initial absorption of water causing the membrane to expand. Before this expansion, drop penetration is slower but, after some time, the membrane has expanded enough to allow the drop to penetrate faster. Thus, whether dry or after absorbing some water, the CS membranes are capable of quickly absorbing water. It was not possible to make a comparison with results in the literature, as no studies were found in which water penetration times or rates were determined for commercial WDs.

#### 3.1.4. Moisture Vapor Transmission Rate

When used as WDs, membranes must absorb exudate and release it in the form of vapor to the outside environment, to prevent saturation with exudate. This ability can be quantified by measuring the MVTR across the membranes. The MVTR was determined in two ways: (i) with dry samples in contact with water vapor and (ii) with dry samples in contact with water. The latter method is more relevant for WDs, as WDs are in direct contact with exudate. The MVTR was also determined for samples exposed to water vapor, as this is a method that has also been employed with WDs (e.g., [70]). The average values of MVTR obtained were 34,400 ± 5400 g H_2_O/m^2^/24 h, for samples in contact with water, and 7470 ± 393 g H_2_O/m^2^/24 h, for samples in contact with water vapor (Table 1). In both cases, the obtained values were higher than values found in the literature for commercial WDs (sample in contact with water: 830 to 12,570 g H_2_O/m^2^/24 h; sample in contact with water vapor: 80 to 2838 g H_2_O/m^2^/24; [70,71]). Thus, the prepared CS membranes had a high ability to release water by evaporation, although extremely high evaporation rates could lead to undesirable wound dehydration.

#### 3.1.5. Thermogravimetric Analysis

**Figure 6:**
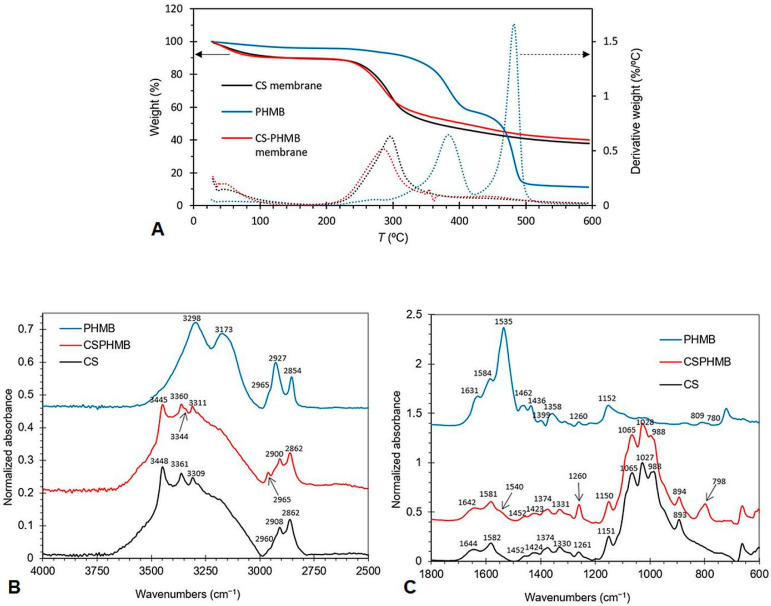
TGA/DTG thermograms and FTIR spectra of CS, PHMB and CS membranes loaded with PHMB. (**A**) TGA (full lines) and DTG (dotted lines) thermograms of CS membranes, CS membranes loaded with PHMB and of PHMB. The TGA and DTG thermograms of the same sample have the same color. (**B**) Normalized FTIR spectra of CS (powder), PHMB (powder) and PHMB-loaded CS membranes (CS + PHMB) in the high-frequency region. (**C**) Normalized FTIR spectra of CS (powder), PHMB (powder) and PHMB-loaded CS membranes (CS + PHMB) in the low-frequency region.

In an attempt to understand whether the addition of PHMB to CS membranes affects the interactions between the polymeric chains of CS, the TGA and DTG thermograms of unloaded and PHMB-loaded CS membranes and of PHMB were obtained (Figure 6A). The values of the extrapolated onset (*T*_o_) and endset (*T*_e_) temperatures, as well as of the inflection temperature (*T*_i_), for both the main transition (transition with the greatest mass loss) and the secondary transition, are presented in Table 2. All curves started with a small mass loss at around 100 °C, which is attributed to the evaporation of residual water retained in the membranes. The thermogram of CS membranes loaded with PHMB was very similar to that of the unloaded CS membranes, but different from that of PHMB, owing to the fact that CS is the predominant component in the PHMB-loaded membranes. For the unloaded CS membrane, only one transition was observed, with a *T*_i_ of 296 °C. The PHMB-loaded membrane showed two transitions: a first transition (main transition) with a *T*_i_ of 285 °C, where the greatest mass loss occurred, and a second, faint transition, with a *T*_i_ of 449 °C, detected in the DTG thermogram. This second transition, which is only present in membranes loaded with PHMB, was close to one of the transitions of PHMB, suggesting that PHMB is present in the membranes. The unloaded CS membrane exhibits *T*_o_ (263 °C) and *T*_i_ (296 °C) values comparable to those of CS membranes found in the literature [72]. When comparing the thermograms of PHMB-loaded and unloaded membranes, the curve of the PHMB-loaded membrane is slightly displaced to the left, showing *T*_o_, *T*_i_ and *T*_e_ values lower than those of the unloaded membrane. This shift to lower temperatures may indicate a decrease in the interactions between the CS polymeric chains caused by the presence of PHMB, suggesting that PHMB may have penetrated the CS network, disturbing the interactions between the CS polymeric chains.

#### 3.1.6. Fourier Transform Infrared Spectroscopy

In order to further confirm the presence of PHMB in the membranes, FTIR spectra were obtained (Figure 6B,C), with the attributions of the main spectral bands presented in Table 3. In the high-frequency region (Figure 6B), two distinct regions were present: (i) the 3750–3000 cm^−1^ region, corresponding to the stretching vibrations of the hydroxyl (OH) and amine (NH) groups, and (ii) the 3000–2800 cm^−1^ region, corresponding to the C–H stretching vibrations of the methine (CH), methylene (CH_2_) and methyl (CH_3_) groups. In the first region, bands centered between 3448 and 3361 cm^−1^ were assigned to the stretching of OH groups in CS and those centered between 3344 and 3173 cm^−1^, to the stretching of the NH bond in both CS and PHMB. In the 3000–2800 cm^−1^ region, the bands centered between 2961 and 2854 cm^−1^ were assigned to the CH stretching in CH, CH_2_ and CH_3_ groups. The presence of methyl groups in the spectra of CS is indicative of the existence of *N*-acetylglucosamine units, as methyl groups do not exist in either PHMB or fully deacetylated CS. This is expected, since the CS employed in this study was not fully deacetylated. A comparison of the FTIR spectra of PHMB-loaded CS membranes and unloaded CS membranes reveals the presence of two new small bands centered at 3344 and 2961 cm^−1^ in the spectrum of the PHMB-loaded CS membrane. The second band is coincident with a PHMB band, while the first band is coincident with a shoulder of a major PHMB band. Thus, the occurrence of these bands in the PHMB-loaded CS membrane suggests the presence of PHMB in the membrane.

In the low-frequency region (Figure 6C and Table 3), two bands centered at 1644 and 1581 cm^−1^ occur in the CS spectrum, assigned to the amide I (mainly due to C=O stretching) and amide II (due to N–H bending and C–N stretching) bands, respectively. These bands arise from *N*-acetylglucosamine units still present in CS. CS also presents bands centered (i) at 1424 and 1374 cm^−1^, assigned to CH vibrations of CH_2_ and CH_3_ groups, (ii) between 1330 and 1261 cm^−1^, assigned to amide III vibrations (coupled C-N stretching and NH bending), CH_2_ wagging and a NHCO vibration, (iii) between 1151 and 1027 cm^−1^, due to skeletal C–O–C and C–N stretching vibrations, and (iv) at 893 cm^−1^, attributed to C–N stretching vibrations and vibrations of the saccharide structure. In this low frequency region, the PHMB spectrum exhibited bands centered at 1631, 1584, 1535, 1463, 1152 cm^−1^, and 811 and 792 cm^−1^, attributed to NH deformation or C=N stretching; NH^+^ bending; NH bending; C=N stretching; C–N stretching and H–N–C bending; and NH_2_ rocking and NH wagging, respectively. A comparison of the spectra of PHMB-loaded CS membranes and unloaded membranes showed three new small bands at 1540, 1260 and 798 cm^−1^. These bands are coincident or close to bands present in the PHMB spectrum but not in the CS spectrum, confirming the presence of PHMB in the PHMB-loaded membrane.

#### 3.1.7. PHMB Loading

To select the best PHMB concentration for drug loading in the CS membranes, the membranes were loaded by soaking in PHMB solutions with concentrations of 0.1 mg/mL, 1 mg/mL, 2 mg/mL or 5 mg/mL of PBS, at 34 °C and for 24 h, under shaking at 100 rpm. The selected temperature (34 °C; a temperature within the range of temperatures found in surface wounds [84,85,86]) was the same employed in the drug release studies, so that the membranes would not expand or contract during the initial phase of the drug release studies. When increasing the concentration of PHMB from 0.1 mg/mL to 1 mg/mL, a slight increase in the amount of PHMB loaded from 5.1 to 8 µg PHMB/mg of dry membrane occurred (Table 4). However, this difference was not statistically significant. When the concentration of PHMB was increased to 2 mg/mL, the amount of loaded PHMB increased to 23.5 µg PHMB/mg of dry membrane and, when further increased to 5 mg/mL, an additional increase was achieved (66 µg PHMB/mg of dry membrane). This increase in drug loading with drug concentration in the soaking solution was expected, as PHMB loading is expected to be mainly governed by diffusion of PHMB from the solution into the membrane interior through a PHMB concentration gradient. When comparing with similar studies in which the loaded amounts of PHMB were reported, our best 24 h loading results were slightly below the best results of Bueno et al. (79 µg PHMB/mg of dry membrane [45]), although it is not clear which PHMB concentration was employed, and higher than the best results of Massarelli et al. (23 µg PHMB/mg of dry membrane [50]).

The use of sterilized membranes is critical when considering their use in WDs. However, sterilization can alter the membrane and/or drug, as well as the drug release kinetics. The sterilization method selected in this study was autoclaving, since it is a readily available sterilization method and it has been employed in the sterilization of some commercially available AMDs (e.g., Excilon^TM^ AMD, from Cardinal Health, Dublin, OH, USA). To prevent membrane deformation, the membranes were sterilized while immersed in a liquid medium. As PHMB-loaded membranes autoclaved in water or PBS would lose some of the loaded PHMB, unloaded membranes were autoclaved while immersed in a PHMB solution. This allowed the membranes to be simultaneously loaded with PHMB and sterilized. The sterilized membranes did not dissolve, their hydrated thickness decreased to ca. 770 µm (Table 1) and, apart from becoming pale yellow, they did not show any major visual or handling differences in relation to unsterilized membranes (Figure 4A). The yellow tint was likely due to occurrence of the Maillard reaction [87,88]. This reaction occurs between the carbonyl groups at the reducing end of CS and its amino groups, forming imines. Due to subsequent reactions of combination, degradation and of structural rearrangements, the imines that form originate melanoidins, which have a yellow/brown color. This process also results in covalent crosslinking of the CS chains, that can result in increased mechanical stability. Cross-sections of the membranes loaded with PHMB and sterilized by autoclaving in a PHMB solution observed by SEM (Figure 4D) did not differ significantly from cross-sections of unsterilized membranes (Figure 4C), suggesting that the sterilization process did not significantly affect the internal morphology of the membranes.

When compared to loading of PHMB for 24 h at 34 °C, loading during autoclaving resulted in a significant increase in the loaded PHMB (from 66 to 111 µg PHMB/mg of dry membrane; Table 4), which is higher than in the above-mentioned comparable reports [45,50]. When drug loading was allowed to occur for a further 24 h at 34 °C after this loading/autoclaving step, no statistically significant difference in drug loading was obtained in relation to the membranes loaded/autoclaved only (Table 4).

#### 3.1.8. PHMB Release

As only one face of an AMD contacts the wound bed and releases the antibacterial agent, the kinetics of PHMB release from a single face of the membranes was studied. The CS membranes loaded with PHMB were mounted between the two chambers of Franz diffusion cells (Figure 3). The donor chamber was empty, while the receptor chamber contained an exudate model. As such, only the underside of the membrane contacted the exudate model and released PHMB. The exudate model employed was PBS at 34 °C and at a pH of 7.4. PBS is a solution that allows to minimally simulate the biological fluids and has been employed in preliminary drug release studies of membranes to be used as WDs [43]. The pH and temperature employed were within the range found in wounds [84,85,86,89,90].

**Figure 7:**
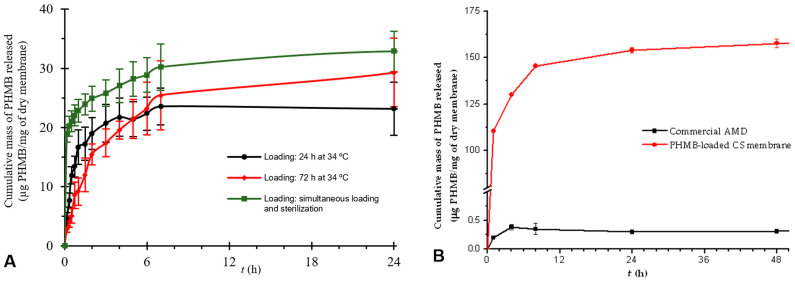
(**A**) Drug release curves of CS membranes loaded by soaking in a 5 mg/mL PHMB solution under different conditions—at 34 °C for 24 h (black line), at 34 °C for 72 h (red line) and during sterilization by autoclaving (green line)—assayed in Franz diffusion cells. (**B**) Drug release curves of a commercial AMD and of the CS membranes loaded with PHMB during sterilization by autoclaving, assayed in batch. Results are expressed as means ± SD (*n* = 3).

In a first study, three membranes in the form of discs were loaded by soaking in a 5 mg/mL solution of PHMB for 24 h at 34 °C. The release of PHMB lasted approximately 7 h (time elapsed until the start of the plateau of the curve), with most PHMB released in the first few hours (Figure 7A). When equilibrium (plateau) was attained, a drug mass of ca. 24 µg of PHMB/mg of dry membrane was released, which is equivalent to ca. 237 µg of PHMB/cm^2^ of membrane. An increase in the drug loading duration from 24 to 72 h resulted in a drug release curve close to that of drug loading for 24 h (Figure 7A). Both the PHMB release duration (ca. 7 h) and the mass of PHMB released when equilibrium was attained (ca. 27 µg of PHMB/mg of dry membrane, equivalent to 345 µg of PHMB/cm^2^ of membrane) were also similar. As such, both the release duration and the mass of PHMB released when equilibrium was attained were not significantly altered by a 48 h increase in the drug loading duration. When PHMB was loaded by soaking also in a 5 mg/mL solution but during sterilization by autoclaving, both the drug release duration and the mass of drug released when equilibrium was attained were comparable to the 24 and 72 h loading studies (ca. 7 h and ca. 30 µg of PHMB/mg of dry membrane, equivalent to 348 µg of PHMB/cm^2^ of membrane; Figure 7A). The mass of released PHMB when equilibrium was attained, although slightly superior, was also close to the values obtained in the 72 h loading study and no statistically significant difference was found between them. Thus, sterilization by autoclaving did not have a major effect on the release kinetics of the PHMB loaded in this type of membrane. This can be seen as advantageous, since (i) it allows obtaining drug release kinetics comparable to that obtained with a much longer loading time (72 h at 34 °C) and (ii) two manufacturing steps—drug loading and sterilization—are combined into one. However, it was not clear whether the drug release duration was adequate for the intended application, which would be at least 24 h (considering a typical daily WD change), or whether the amount of drug released is sufficient to cause bacterial growth inhibition or death. This is because, as the wound model employed—Franz diffusion cell containing 14.55 mL of PBS at 34 °C and pH 7.4—is very far from the in vivo wound conditions, both the in vivo drug release duration and its amount will be different. In fact, the exudate model employed differs extensively from exudate in terms of, in particular, volume, composition, viscosity and turnover rate [91]. As the release medium volume was much larger than the exudate volume in a wound and as, in a non-erodible drug release system, a drug concentration gradient between the drug-containing matrix and the release medium is the driving force for drug release, the maximum drug release rate will occur under infinite sink conditions. Infinite sink conditions occur when the released drug accumulating in the medium is considered negligible and will not affect the dissolution of further drug molecules that are released. In pharmaceutical sciences, it is considered that this is attained when the volume of the release medium is, at least, three to ten times the saturation volume for the drug under study [92]. When these conditions are not attained, finite sink conditions (also designated non-sink conditions) will be present, and drug release will be delayed because of drug accumulation in the release medium. As infinite sink conditions are unlikely to occur in a wound [93], in particular, when covered with a WD that absorbs exudate, the use of much larger release medium volumes will underestimate the drug release duration. A literature search did not find any reports of drug release assays in which commercial AMDs were evaluated using Franz diffusion cells. The only research study employing Franz diffusion cells and PHMB-loaded membranes intended to be used in AMDs, although not sterilized after drug loading, and in which sufficient detail was provided, showed a drug release duration of 48 h and a cumulative drug release when equilibrium was attained of ca. 5 µg of PHMB/mg of dry membrane [50]. However, a direct comparison is precluded due to lack of information concerning the release medium volume employed.

#### 3.1.9. Assay of a Commercial PHMB-Releasing Antimicrobial Dressing

Since the volume used in the Franz diffusion cell model was much larger than the volume of exudate present in any type of wound, an assessment of the suitability of the drug release kinetics for the intended application was not possible with this model. A preliminary validation of the suitability of the prepared membranes for use in AMDs was then obtained through comparing their drug release curves to that of a PHMB-releasing commercial AMD assayed under the same conditions. The commercial AMD selected was Telfa^®^ AMD^TM^, a cotton-based AMD that releases PHMB [94]. For that purpose, a sample of Telfa^®^ AMD^TM^ was assayed in Franz diffusion cells under the same conditions as the prepared membranes. However, it was not possible to obtain a drug release curve, as the released PHMB was too diluted in the large volume of PBS present in the acceptor chamber of the cell. As Franz diffusion cells with a much lower volume/opening area ratio were not available, an alternative assay was devised so that a much lower medium volume/sample area ratio could be employed. This drug release assay was carried out (i) in batch (i.e., with the samples fully immersed in the release medium), (ii) in a volume of 2 mL of PBS under magnetic stirring, a volume much closer to the in vivo exudate volume than when employing Franz diffusion cells, and (iii) under a turnover rate of 1.6 mL/day (calculated from the aliquot volumes collected and replaced by PBS per 24 h), equivalent to 5 mL/10 cm^2^/24 h, which is within the range of reported values for exudate turnover in different types of wounds [91,95,96]. The commercial AMD showed a drug release duration of ca. 4 h, while the CS membranes loaded with PHMB during sterilization by autoclaving showed a slightly higher drug release duration (ca. 6 h; Figure 7B). This result indicates that the PHMB-loaded CS membranes release PHMB at a rate that is comparable to that of a commercial AMD when tested in the same mode. This suggests that they may be suitable for use in AMDs. However, the drug release duration obtained for Telfa^®^ AMD^TM^ in this assay (ca. 4 h) is much shorter than its known antimicrobial effect duration (up to 3 days [97]). This indicates that the conditions under which this drug release assay was carried out are not yet representative of the in vivo conditions. When equilibrium was attained, the CS membrane released significantly more PHMB (ca. 150 µg of PHMB/mg of dry membrane; Figure 7B) than the commercial AMD (ca. 3 µg of PHMB/mg of dry membrane), which is favorable for the intended application, although it must be verified whether the amount of released PHMB does not cause cytotoxicity.

### 3.2. Biological Characterization

#### 3.2.1. Antibacterial Activity of PHMB in Solution

Before evaluating the antibacterial activity of CS membranes loaded with PHMB by soaking in a 5 mg/mL PHMB solution during autoclaving, the MICs of PHMB solutions sterilized by membrane filtration or by autoclaving were compared for representative bacterial strains commonly present in wounds [98]: *E. coli* and *P. aeruginosa*, as Gram-negative bacteria, and *S. aureus*, as Gram-positive. The goal of this determination was to verify whether autoclaving affected the antibacterial activity of PHMB in solution. For both *E. coli* and *S. aureus*, the MIC values obtained were 3.1 µg/mL for the solutions sterilized by either method (Table 5). For *P. aeruginosa*, the MIC values were also equal but a higher value of 12.5 µg/mL was obtained. This higher value was expected, since *P. aeruginosa* is often more resistant to antibacterial agents and antibiotics, due to its intrinsic resistance mechanisms [99]. Thus, sterilization by autoclaving did not affect the antibacterial activity of PHMB. The obtained MIC values were slightly above values published in the literature, although the literature studies employed different bacterial strains [29,100,101].

#### 3.2.2. Antibacterial Activity of the Membranes on Bacterial Suspensions

**Figure 8:**
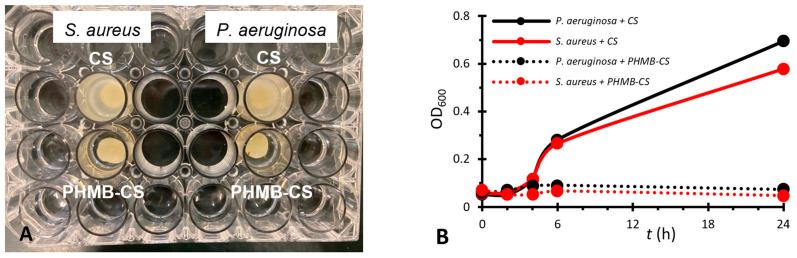
Photographs of cell culture plates (**A**) and growth curves (**B**) of bacterial suspensions of *S. aureus* and *P. aeruginosa* in contact with autoclaved, unloaded CS membranes (CS) and with CS membranes loaded with PHMB by soaking during autoclaving (PHMB-CS). (**A**) Cell culture plates 24 h after the start of the assay. (**B**) Growth curves of the bacterial suspensions.

The CS membranes loaded with PHMB were placed in bacterial suspensions with the aim of verifying whether they were capable of inhibiting bacterial growth in suspension. Unloaded, autoclaved CS membranes were also evaluated. The bacterial cultures selected were a difficult to eradicate, Gram-negative bacterium *(P. aeruginosa*) and a Gram-positive bacterium (*S. aureus*). The occurrence of bacterial growth inhibition could be visually identified in the case of membranes with PHMB, but not in the cases of membranes without PHMB (Figure 8A). The bacterial growth curves showed that the CS membranes not containing PHMB did not cause growth inhibition (Figure 8B). As this assay was carried out in LB medium, which has a pH of 7, and as the antibacterial activity of CS depends on the existence of protonated amino groups [15], most of its amino groups will be unprotonated at this pH (p*K*_a_ of amino groups in CS: 6.4–6.5 [102]).

For the CS membranes loaded with PHMB, less bacterial growth and an exponential phase occurring later than for membranes without PHMB would be expected. However, the results obtained were superior, with an almost complete inhibition of bacterial growth (Figure 8B), which indicates that the PHMB-loaded CS membranes have high antibacterial activity.

#### 3.2.3. Antibacterial Activity of the Membranes on Bacterial Cultures on Agar

**Figure 9:**
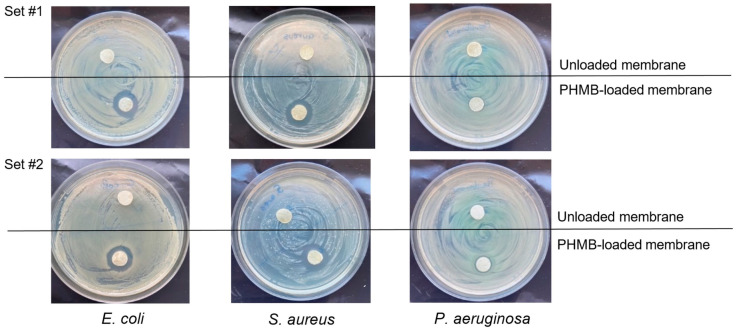
Photographs of the inhibition halos on agar plates containing bacterial cultures of *E. coli*, *S. aureus* and *P. aeruginosa* after 24 h of direct contact with PHMB-loaded CS membranes by soaking during autoclaving (PHMB-loaded membranes) and with CS membranes autoclaved in PBS (unloaded membranes), in duplicate.

The antibacterial activity of the developed membranes was studied through the agar diffusion test, in which CS membranes loaded with PHMB during sterilization by autoclaving were placed in direct contact with bacterial cultures of *E. coli*, *S. aureus* and *P. aeruginosa* on agar plates, i.e., bacterial cultures growing on a surface, as occurs in wounds. For each strain, CS membranes loaded with PHMB by soaking during autoclaving and unloaded membranes autoclaved in PBS were studied. Figure 9 shows photographs of the agar plates containing the membranes and the respective inhibition halos after 24 h of direct contact between the membranes and the bacterial cultures. The unloaded membranes did not produce an inhibition halo. As this assay was carried out in MH medium, which has a pH of 7.3, most of CS’s amino groups will be unprotonated and CS will have low antibacterial activity. In the PHMB-loaded membranes, the presence of inhibition halos was observed for all tested bacteria (Figure 9 and Table 5), indicating that PHMB diffusing from the sample caused inhibition of bacterial growth of Gram-negative and Gram-positive bacteria usually present in infected wounds. A smaller inhibition halo was observed for *P. aeruginosa* compared to *E. coli* and *S. aureus* due to its already mentioned higher resistance to antimicrobial agents [99], making growth inhibition more difficult. However, even for this difficult-to-eradicate species, the membranes showed effective growth inhibition.

#### 3.2.4. Blood Clotting Activity

WDs that promote blood clotting when applied to wounds are advantageous, since they prevent bleeding. Therefore, the potential of the PHMB-loaded CS membranes in inducing blood clotting was assessed. The assay selected involved the in vitro determination of the degree of thrombosis after contact between blood and CS membranes loaded with PHMB during autoclaving or membranes not loaded with PHMB but autoclaved in a PBS solution. Both types of membranes promoted blood clotting (Table 6), although the degree of thrombosis induced by unloaded CS membranes (52%) was higher than that induced by PHMB-loaded CS membranes (28%). Thus, it was confirmed that CS alone is capable of inducing blood clotting [16]. CS causes agglutination of erythrocytes through binding to their membranes, possibly via ionic interactions between CS’s positively charged amino groups and negatively charged residues on the cell membrane [103]. It also causes platelet activation through an increase in the expression of platelet’s GPIIb/IIIa membrane receptor [104]. However, when loaded with PHMB, its blood clotting activity decreased, in spite of the fact that, at physiological pH, PHMB is a positively charged polymer (p*K*_a_ of ca. 13 [63]) that could also promote the agglutination of erythrocytes. The observed decrease in the blood clotting capacity of the PHMB-loaded CS membranes may be owed to less availability of the positively charged amine and biguanide groups, due to interchain interactions.

## 4. Conclusions

Highly porous membranes made of neat CS could be prepared using cryogelation. They exhibited high water swelling capacity, fast water absorption and high water vapor transmission. The best loading method combined simultaneous drug loading by soaking in a PHMB solution with sterilization by autoclaving, resulting in sterile, drug-loaded membranes. The drug release kinetics of these membranes was comparable to those of a commercial AMD that also released PHMB assayed under the same conditions. The membranes showed procoagulant activity and high antibacterial activity against a set of Gram-negative and Gram-positive bacterial species selected among those commonly present in infected wounds, both in contact with bacterial suspensions and in direct contact with the bacterial culture growing on a surface. These results suggest that these membranes may be suitable for use in AMDs, although a subsequent biocompatibility evaluation must follow this study.

## Figures and Tables

**Table 1:** Thickness, swelling capacity, water contact angle, drop penetration time and rate and moisture vapor transmission rate (MVTR) of the CS membranes. Results are expressed as mean ± SD.

Property	Dry Membrane	Hydrated Membrane
Thickness (µm, *n* = 3)	220 ± 24	1200 ± 235
After autoclaving	– *	770 ± 100
Swelling capacity (%; *n* = 3)	748 ± 23	*–* *
Water contact angle (°; *n* = 3)	97 ± 2	82 ± 6
Drop penetration time (s; *n* = 3)	11 ± 4	4.1 ± 0.6
Drop penetration rate(s) (mm/s; *n* = 4–5)	0.02 ± 0.01; 0.10 ± 0.02	0.24 ± 0.05
MVTR (g H_2_O/m^2^/24 h)		
Contact with water vapor (*n* = 6)	7470 ± 393	– *
Contact with water (*n* = 6)	34,400 ± 5400	– *

* Not applicable.

**Table 2:** Temperatures of the transitions present in the TGA/DTG thermograms of CS membranes, CS membranes loaded with PHMB and PHMB. *T*_o_—extrapolated onset temperature; *T*_i_—extrapolated inflexion temperature; *T*_e_—extrapolated endset temperature.

Sample	Main Transition				Secondary Transition		
	*T*_o_ (°C)	*T*_i_ (°C)	*T*_e_ (°C)		*T*_o_ (°C)	*T*_i_ (°C)	*T*_e_ (°C)
Unloaded CS membrane	263	296	333		– *	– *	– *
PHMB	469	482	491		357	383	401
PHMB-loaded CS membrane	250	285	308		420	449	476

* Secondary transition not present.

**Table 3:** Assignments of the main bands in the FTIR spectra of CS and PHMB-loaded CS membranes (CS + PHMB) and of PHMB (powder).

Band Position (cm^−1^)			Assignment *	References *
CS	CS + PHMB	PHMB		
3448	3443	–	OH stretching	[73,74]
3361	3362			
–	3344	–	NH stretching	[75]
3309	3311			[74]
–	–	3298		[76,77]
–	–	3173	Symmetric NH stretching	[76]
2960	2961	2959	CH asymmetric stretching in CH_3_	[78]
2908	2908	–	CH stretching in CH_3_, CH_2_ and CH	[74,79]; [75,80]
–	–	2927		
2862	2861	2854		
1644	1642	1631	Amide I (CS); NH deformation or C=N stretching (PHMB)	[79,81]; [76]
1581	1581	1584	Amide II (CS); NH^+^ bending (PHMB)	[74]; [82]
–	1540	1535	NH bending	[83]
	–	1463	C=N stretching	[83]
1424	1423	–	CH_2_ bending	[74]
1374	1374		CH_3_ symmetric bending	[81]
1330	1331	–	Amide III and CH_2_ wagging	[74]
1261	1260		NHCO vibration	[79]
1151	1150	1152	C–O stretching in C–O–C vibrations and C–N stretching (CS); C–N stretching and H–N–C bending (PHMB)	[73,79,81]; [77,82]
1065	1065	–		
1027	1028			
893	894	–	CN vibration and vibration of the saccharide structure	[73,79]
–	798	811 + 792	NH_2_ rocking and NH wagging	[76]

* Assignments and references related to CS are separated from those related to PHMB by a semi-colon.

**Table 4:** Comparison of the amount of PHMB loaded by soaking CS membranes in PHMB solutions with a concentration of 0.1, 1, 2 and 5 mg/mL in PBS, under different conditions.

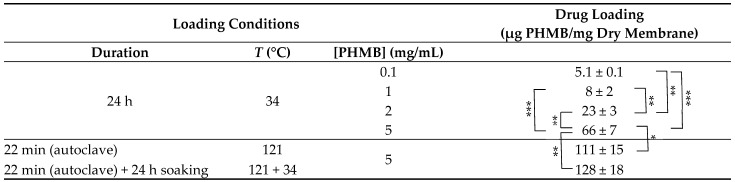			

* *p* < 0.05; ** *p* < 0.01; *** *p* < 0.001 (ANOVA, followed by Tukey’s HSD test, at a confidence level of 95%). Results are expressed as mean ± SD (*n* = 3).

**Table 5:** Minimum inhibitory concentrations (MIC) of PHMB for *E. coli*, *S. aureus* and *P. aeruginosa* stains obtained with PHMB solutions sterilized by membrane filtration or by autoclaving and diameters of the inhibition halos obtained in the diffusion on agar assay of CS membranes loaded with PHMB by soaking during autoclaving (direct contact with bacterial cultures on agar plates). Disc diameter: 1 cm. The assays were performed in duplicate. When different values were obtained in the duplicates, the mean ± SD was represented.

Bacteria	MIC		Inhibition Halo Diameter (cm)
	PHMB Solution Sterilized by Membrane Filtration (μg/mL)	PHMB Solution Sterilized by Autoclaving (μg/mL)	
*E. coli*	3.1 ^a^	3.1 ^a^	1.8 ± 0.2
*S. aureus*	3.1 ^a^	3.1 ^a^	1.9 ± 0.1
*P. aeruginosa*	12.5 ^a^	12.5 ^a^	1.2 ^a^

^a^ The same value was obtained in the duplicates.

**Table 6:** Thrombosis degree of CS membranes loaded by soaking in a 5 mg/mL PHMB solution in PBS during autoclaving and of unloaded, autoclaved CS membranes (autoclaved in the presence of PBS).

Membrane Type	Thrombosis Degree (%)
Unloaded	54 ± 14 *
Loaded with PHMB by soaking/autoclaving	28 ± 2 *

* *p* < 0.05 (Student’s *t* test, at a confidence level of 95%; *n* = 3–4). Results are expressed as mean ± SD.

## Data Availability

The data presented in this study are available on request from the corresponding author.

## References

[1] Lazarus G.S., Cooper D.M., Knighton D.R., Margolis D.J., Pecoraro R.E., Rodeheaver G., Robson M.C. (1994). Definitions and guidelines for assessment of wounds and evaluation of healing. JAMA Dermatol..

[2] Rodrigues M., Kosaric N., Bonham C.A., Gurtner G.C. (2019). Wound healing: A cellular perspective. Physiol. Rev..

[3] Wilkinson H.N., Hardman M.J. (2020). Wound healing: Cellular mechanisms and pathological outcomes. Open Biol..

[4] Velnar T., Bailey T., Smrkolj V. (2009). The wound healing process: An overview of the cellular and molecular mechanisms. J. Int. Med. Res..

[5] Sen C.K. (2021). Human wound and its burden: Updated 2020 compendium of estimates. Adv. Wound Care.

[6] Kunjikuttan R.V.P., Jayasree A., Biswas R., Jayakumar R. (2016). Recent developments in drug-eluting dressings for the treatment of chronic wounds. Exp. Opin. Drug Deliv..

[7] Johnson N.R., Wang Y. (2015). Drug delivery systems for wound healing. Curr. Pharm. Biotechnol..

[8] Settipalli S. A Robust Market Rich with Opportunities: Advanced Wound Dressings. https://www.pm360online.com/a-robust-market-rich-with-opportunities-advanced-wound-dressings/.

[9] WoundSource Woundsource: The Kestrel Wound Product Sourcebook. https://www.woundsource.com/.

[10] Ji M., Li J., Wang Y., Li F., Man J., Li J., Zhang C., Peng S., Wang S. (2022). Advances in chitosan-based wound dressings: Modifications, fabrications, applications and prospects. Carbohydr. Polym..

[11] Parhi R. (2020). Drug delivery applications of chitin and chitosan: A review. Environm. Chem. Lett..

[12] Mercy H.E., Halim A.M.A., Hussein A.A. (2012). Chitosan-derivatives as hemostatic agents: Their role in tissue regeneration. Regen. Res..

[13] Kou S., Peters L., Mucalo M. (2022). Chitosan: A review of molecular structure, bioactivities and interactions with the human body and micro-organisms. Carbohydr. Polym..

[14] Maliki S., Sharma G., Kumar A., Moral-Zamorano M., Moradi O., Baselga J., Stadler F.J., García-Peñas A. (2022). Chitosan as a tool for sustainable development: A mini review. Polymers.

[15] Li J., Zhuang S. (2020). Antibacterial activity of chitosan and its derivatives and their interaction mechanism with bacteria: Current state and perspectives. Eur. Polym. J..

[16] Okamoto Y., Yano R., Miyatake K., Tomohiro I., Shigemasa Y., Minami S. (2003). Effects of chitin and chitosan on blood coagulation. Carbohydr. Polym..

[17] Patrulea V., Ostafe V., Borchard G., Jordan O. (2015). Chitosan as a starting material for wound healing applications. Eur. J. Pharm. Biopharm..

[18] Dai T., Tanaka M., Huang Y.Y., Hamblin M.R. (2011). Chitosan preparations for wounds and burns: Antimicrobial and wound-healing effects. Expert Rev. Anti-Infect. Ther..

[19] Grzybek P., Jakubski Ł., Dudek G. (2022). Neat chitosan porous materials: A review of preparation, structure characterization and application. Int. J. Mol. Sci..

[20] Hsieh C.-Y., Tsai S.-P., Ho M.-H., Wang D.-M., Liu C.-E., Hsieh C.-H., Tseng H.-C., Hsieh H.-J. (2007). Analysis of freeze-gelation and cross-linking processes for preparing porous chitosan scaffolds. Carbohydr. Polym..

[21] Kaehn K. (2010). Polihexanide: A safe and highly effective biocide. Skin. Pharmacol. Physiol..

[22] Kramer A., Dissemond J., Kim S., Willy C., Mayer D., Papke R., Tuchmann F., Assadian O. (2018). Consensus on wound antisepsis: Update 2018. Skin. Pharmacol. Physiol..

[23] Bernauer U., Bodin L., Celleno L., Chaudhry Q.M., Coenraads P.-J., Dusinska M., Duus-Johansen J., Ezendam J., Gaffet E., Galli C.L. (2016). SCCS Opinion on Polyaminopropyl Biguanide (PHMB)—Submission III, SCCS/1581/16, Preliminary Version of 23 December 2016.

[24] Gao Y., Cranston R. (2008). Recent advances in antimicrobial treatments of textiles. Text. Res. J..

[25] Magina S., Santos M.D., Ferra J., Cruz P., Portugal I., Evtuguin D. (2016). High pressure laminates with antimicrobial properties. Materials.

[26] Chivu A., Chindera K., Mendes G., An A., Davidson B., Good L., Song W. (2021). Cellular gene delivery via poly(hexamethylene biguanide)/pDNA self-assembled nanoparticles. Eur. J. Pharm. Biopharm..

[27] Wang W.-Y., Chiou J.-C., Chen W.-X., Yu J.-L., Kan C.-W. (2022). A salt-free, zero-discharge and dyebath-recyclable circular coloration technology based on cationic polyelectrolyte complex for cotton fabric dyeing. Cellulose.

[28] Kazanskiy N.L., Butt M.A., Khonina S.N. (2021). Carbon dioxide gas sensor based on polyhexamethylene biguanide polymer deposited on silicon nano-cylinders metasurface. Sensors.

[29] Koburger T., Hübner N.-O., Braun M., Siebert J., Kramer A. (2010). Standardized comparison of antiseptic efficacy of triclosan, PVP–iodine, octenidine dihydrochloride, polyhexanide and chlorhexidine digluconate. J. Antimicrob. Chemother..

[30] Moore K., Gray D. (2007). Using PHMB antimicrobial to prevent wound infection. Wounds UK.

[31] Hübner N.O., Kramer A. (2010). Review on the efficacy, safety and clinical applications of polihexanide, a modern wound antiseptic. Skin. Pharmacol. Physiol..

[32] Larkin D.F., Kilvington S., Dart J.K. (1992). Treatment of Acanthamoeba keratitis with polyhexamethylene biguanide. Ophthalmology.

[33] Valluri S., Fleming T.P., Laycock K.A., Tarle I.S., Goldberg M.A., Garcia-Ferrer F.J., Essary L.R., Pepose J.S. (1997). In vitro and in vivo effects of polyhexamethylene biguanide against herpes simplex virus infection. Cornea.

[34] Krebs F.C., Miller S.R., Ferguson M.L., Labib M., Rando R.F., Wigdahl B. (2005). Polybiguanides, particularly polyethylene hexamethylene biguanide, have activity against human immunodeficiency virus type 1. Biomed. Pharmacother..

[35] Pinto F., Maillard J.-Y., Denyer S.P., McGeechan P. (2010). Polyhexamethylene biguanide exposure leads to viral aggregation. J. Appl. Microbiol..

[36] Kramer A., Roth B., Müller G., Rudolph P., Klöcker N. (2004). Influence of the antiseptic agents polyhexanide and octenidine on FL cells and on healing of experimental superficial aseptic wounds in piglets. A double-blind, randomised, stratified, controlled, parallel-group study. Skin. Pharmacol. Physiol..

[37] Gilbert P., Moore L.E. (2005). Cationic antiseptics: Diversity of action under a common epithet. J. Appl. Microbiol..

[38] Glukhov E., Stark M., Burrows L.L., Deber C.M. (2005). Basis for selectivity of cationic antimicrobial peptides for bacterial versus mammalian membranes. J. Biol. Chem..

[39] Chindera K., Mahato M., Sharma A.K., Horsley H., Kloc-Muniak K., Kamaruzzaman N.F., Kumar S., McFarlane A., Stach J., Bentin T. (2016). The antimicrobial polymer PHMB enters cells and selectively condenses bacterial chromosomes. Sci. Rep..

[40] Wessels S., Ingmer H. (2013). Modes of action of three disinfectant active substances: A review. Regul. Toxicol. Pharmacol..

[41] East G.C., McIntyre J.E., Shao J. (1997). Polybiguanides: Synthesis and characterization of polybiguanides containing hexamethylene groups. Polymer.

[42] Küsters M., Beyer S., Kutscher S., Schlesinger H., Gerhartz M. (2013). Rapid, simple and stability-indicating determination of polyhexamethylene biguanide in liquid and gel-like dosage forms by liquid chromatography with diode-array detection. J. Pharm. Anal..

[43] Guiomar A.J., Urbano A.M. (2022). Polyhexanide-releasing membranes for antimicrobial wound dressings: A critical review. Membranes.

[44] De Paula G.F., Netto G.I., Mattoso L.H.C. (2011). Physical and chemical characterization of poly(hexamethylene biguanide) hydrochloride. Polymers.

[45] Dilamian M., Montazer M., Masoumi J. (2013). Antimicrobial electrospun membranes of chitosan/poly(ethylene oxide) incorporating poly(hexamethylene biguanide) hydrochloride. Carbohydr. Polym..

[46] Bueno C.Z., Moraes A.M. (2018). Influence of the incorporation of the antimicrobial agent polyhexamethylene biguanide on the properties of dense and porous chitosan-alginate membranes. Mater. Sci. Eng. C Mater. Biol. Appl..

[47] Abri S., Ghatpande A.A., Ress J., Barton H.A., Leipzig N.D. (2019). Polyionic complexed antibacterial heparin–chitosan particles for antibiotic delivery. ACS Appl. Bio Mater..

[48] Ni Y., Qian Z., Yin Y., Yuan W., Wu F., Jin T. (2020). Polyvinyl alcohol/chitosan/polyhexamethylene biguanide phase separation system: A potential topical antibacterial formulation with enhanced antimicrobial effect. Molecules.

[49] Ng I.S., Ooi C.W., Liu B.L., Peng C.T., Chiu C.Y., Chang Y.K. (2020). Antibacterial efficacy of chitosan- and poly(hexamethylene biguanide)-immobilized nanofiber membrane. Int. J. Biol. Macromol..

[50] Massarelli E., Silva D., Pimenta A.F.R., Fernandes A.I., Mata J.L.G., Armes H., Salema-Oom M., Saramago B., Serro A.P. (2021). Polyvinyl alcohol/chitosan wound dressings loaded with antiseptics. Int. J. Pharm..

[51] Lin Y.J., Chien B.Y.C., Lee Y.H. (2022). Injectable and thermoresponsive hybrid hydrogel with antibacterial, anti-inflammatory, oxygen transport, and enhanced cell growth activities for improved diabetic wound healing. Eur. Polym. J..

[52] Guo C.A., Zhang J., Feng X.J., Du Z.G., Jiang Y.Z., Shi Y.D., Yang G.H., Tan L. (2022). Polyhexamethylene biguanide chemically modified cotton with desirable hemostatic, inflammation-reducing, intrinsic antibacterial property for infected wound healing. Chin. Chem. Lett..

[53] Lee Y.H., Lin S.J. (2022). Chitosan/pva hetero-composite hydrogel containing antimicrobials, perfluorocarbon nanoemulsions, and growth factor-loaded nanoparticles as a multifunctional dressing for diabetic wound healing: Synthesis, characterization, and in vitro/in vivo evaluation. Pharmaceutics.

[54] Nydrioti E., Saramago B., Serro A.P. (2021). Polyhexanide and chlorhexidine loaded chitosan wound dressings. Ann. Med..

[55] Harrington R.E., Guda T., Lambert B., Martin J., Wagner W.R., Zhang G., Sakiyama-Elbert S.E., Yaszemski M.J. (2020). Sterilization and disinfection of biomaterials for medical devices. Biomaterials Science. An Introduction to Materials in Medicine.

[56] Petersen S., Hussner J., Reske T., Grabow N., Senz V., Begunk R., Arbeiter D., Kroemer H.K., Schmitz K.-P., Meyer zu Schwabedissen H.E. (2013). In vitro study of dual drug-eluting stents with locally focused sirolimus and atorvastatin release. J. Mater. Sci. Mater. Med..

[57] (2002). Test Methods for Primary Wound Dressings. Part 2—Moisture Vapour Transmission Rate of Permeable Film Dressings.

[58] Franz T.J. (1975). Percutaneous absorption on the relevance of in vitro data. J. Investig. Dermatol..

[59] Imai Y., Nose Y. (1972). A new method for evalution of antithrombogenicity of materials. J. Biomed. Mater. Res..

[60] Groth T., Derdau K., Strietzel F., Foerster F., Wolf H. (1992). The haemocompatibility of biomaterials in vitro: Investigations on the mechanism of the whole-blood clot formation test. Altern. Lab. Anim..

[61] Minsart M., Van Vlierberghe S., Dubruel P., Mignon A. (2022). Commercial wound dressings for the treatment of exuding wounds: An in-depth physico-chemical comparative study. Burn. Trauma.

[62] Mason P.E., Neilson G.W., Enderby J.E., Saboungi M.-L., Dempsey C.E., MacKerell A.D., Brady J.W. (2004). The structure of aqueous guanidinium chloride solutions. J. Am. Chem. Soc..

[63] Blackburn R.S., Harvey A., Kettle L.L., Payne J.D., Russell S.J. (2006). Sorption of poly(hexamethylenebiguanide) on cellulose:  Mechanism of binding and molecular recognition. Langmuir.

[64] Zaki A.M., Troisi A., Carbone P. (2016). Unexpected like-charge self-assembly of a biguanide-based antimicrobial polyelectrolyte. J. Chem. Phys. Lett..

[65] Vogler E.A., Morra M. (2001). On the origins of water wetting terminology. Water in Biomaterials Surface Science.

[66] Luo Y., Pan X., Ling Y., Wang X., Sun R. (2014). Facile fabrication of chitosan active film with xylan via direct immersion. Cellulose.

[67] Wenzel R.N. (1936). Resistance of solid surfaces to wetting by water. Ind. Eng. Chem..

[68] Cassie A.B.D., Baxter S. (1944). Wettability of porous surfaces. Trans. Faraday Soc..

[69] Wang Z., Elimelech M., Lin S. (2016). Environmental applications of interfacial materials with special wettability. Environ. Sci. Technol..

[70] Bainbridge P., Browning P., Bernatchez S.F., Blaser C., Hitschmann G. (2021). Comparing test methods for moisture-vapor transmission rate (MVTR) for vascular access transparent semipermeable dressings. J. Vasc. Access.

[71] Zehrer C.L., Holm D., Solfest S.E., Walters S.-A. (2014). A comparison of the in vitro moisture vapour transmission rate and in vivo fluid-handling capacity of six adhesive foam dressings to a newly reformulated adhesive foam dressing. Int. Wound J..

[72] Chen C.-H., Wang F.-Y., Mao C.-F., Yang C.-H. (2007). Studies of chitosan. I. Preparation and characterization of chitosan/poly(vinyl alcohol) blend films. J. Appl. Polym. Sci..

[73] Ibrahim M., Osman O., Mahmoud A.A. (2011). Spectroscopic analyses of cellulose and chitosan: FTIR and modeling approach. J. Comput. Theor. Nanosci..

[74] Mauricio-Sanchez R.A., Salazar R., Luna-Barcenas J.G., Mendoza-Galvan A. (2018). FTIR spectroscopy studies on the spontaneous neutralization of chitosan acetate films by moisture conditioning. Vib. Spectrosc..

[75] Ramasamy S., Muthusamy S., Nagarajan S., Nath A.V., Savarimuthu J.S., Jayaprakash J., Gurunadhan R.M. (2022). Fabrication of collagen with polyhexamethylene biguanide: A potential scaffold for infected wounds. J. Biomed. Mater. Res. Part B Appl. Biomater..

[76] Sheela N.R., Muthu S., Krishnan S.S. (2010). FTIR, FT Raman and UV-visible spectroscopic analysis on metformin hydrochloride. Asian J. Chem..

[77] Rodrigues A.M., Silva S.Y.S., Oliveira M.N., de Oliveira G.C.A., Novais A.L.F., de Paula G.F., Souza D.N., Belo E.A., Gester R., Andrade-Filho T. (2021). Prediction of electronic and vibrational properties of poly(hexamethylene biguanide) hydrochloride: A combined theoretical and experimental investigation. J. Mol. Struct..

[78] Bellamy L.J. (1975). Alkanes. The Infra-Red Spectra of Complex Molecules.

[79] Nga N.K., Chinh H.D., Hong P.T.T., Huy T.Q. (2017). Facile preparation of chitosan films for high performance removal of reactive blue 19 dye from aqueous solution. J. Polym. Environ..

[80] Worsley A., Vassileva K., Tsui J., Song W.H., Good L. (2019). Polyhexamethylene biguanide:Polyurethane blend nanofibrous membranes for wound infection control. Polymers.

[81] Lawrie G., Keen I., Drew B., Chandler-Temple A., Rintoul L., Fredericks P., Grøndahl L. (2007). Interactions between alginate and chitosan biopolymers characterized using FTIR and XPS. Biomacromolecules.

[82] Celik S., Tanıs E. (2022). Toxic potential of poly-hexamethylene biguanide hydrochloride (PHMB): A DFT, AIM and NCI analysis study with solvent effects. Comput. Theor. Chem..

[83] Llorens E., Calderon S., del Valle L.J., Puiggali J. (2015). Polybiguanide (PHMB) loaded in PLA scaffolds displaying high hydrophobic, biocompatibility and antibacterial properties. Mater. Sci. Eng. C Mater. Biol. Appl..

[84] Dini V., Salvo P., Janowska A., Di Francesco F., Barbini A., Romanelli M. (2015). Correlation between wound temperature obtained with an infrared camera and clinical wound bed score in venous leg ulcers. Wounds.

[85] Hellgren L., Vincent J. (1977). Degradation and liquefaction effect of streptokinase-Streptodornase and stabilized trypsin on necroses, crusts of fibrinoid, purulent exudate and clotted blood from leg ulcers. J. Int. Med. Res..

[86] Lamke L.O., Nilsson G.E., Reithner H.L. (1977). The evaporative water loss from burns and the water-vapour permeability of grafts and artificial membranes used in the treatment of burns. Burns.

[87] Baynes J.W. (2005). The Maillard reaction:  Chemistry, biochemistry and implications. J. Am. Chem. Soc..

[88] Leceta I., Guerrero P., de la Caba K. (2013). Functional properties of chitosan-based films. Carbohydr. Polym..

[89] Gethin G., Ivory J.D., Sezgin D., Muller H., O’Connor G., Vellinga A. (2021). What is the “normal” wound bed temperature? A scoping review and new hypothesis. Wound Repair Regen..

[90] Ono S., Imai R., Ida Y., Shibata D., Komiya T., Matsumura H. (2015). Increased wound pH as an indicator of local wound infection in second degree burns. Burns.

[91] World Union of Wound Healing Societies (WUWHS) (2019). Consensus Document. Wound Exudate: Effective Assessment and Management.

[92] European Directorate for the Quality of Medicines & Health Care (EDQM) (2008). European Pharmacopoeia 6.0. Dissolution Test for Solid Dosage Forms (01/2008:20903).

[93] Steffansen B., Herping S.P.K. (2008). Novel wound models for characterizing ibuprofen release from foam dressings. Int. J. Pharm..

[94] Cardinal Health Cardinal Health™ Telfa™ AMD Antimicrobial Non-Adherent Dry Dressings. https://www.cardinalhealth.com/en/product-solutions/medical/skin-and-wound-management/traditional-wound-care/non-adherent-dry-dressings/telfa-amd-dressings.html.

[95] Cutting K.F. (2003). Wound exudate: Composition and functions. Br. J. Community Nurs..

[96] Thomas S.T., Rajendran S. (2009). Testing dressings and wound management materials. Advanced Textiles for Wound Care.

[97] WoundSource Woundsource Product Guide—Telfa AMD Antimicrobial Dressings. https://www.woundsource.com/product/telfa-amd-antimicrobial-dressings.

[98] Puca V., Marulli R.Z., Grande R., Vitale I., Niro A., Molinaro G., Prezioso S., Muraro R., Di Giovanni P. (2021). Microbial species isolated from infected wounds and antimicrobial resistance analysis: Data emerging from a three-years retrospective study. Antibiotics.

[99] Lambert P.A. (2002). Mechanisms of antibiotic resistance in *Pseudomonas aeruginosa*. J. R. Soc. Med..

[100] Fabry W.H.K., Kock H.J., Vahlensieck W. (2014). Activity of the antiseptic polyhexanide against Gram-negative bacteria. Microb. Drug. Resist..

[101] Fabry W., Kock H.J. (2014). In-vitro activity of polyhexanide alone and in combination with antibiotics against *Staphylococcus aureus*. J. Hosp. Infect..

[102] Wang Q.Z., Chen X.G., Liu N., Wang S.X., Liu C.S., Meng X.H., Liu C.G. (2006). Protonation constants of chitosan with different molecular weight and degree of deacetylation. Carbohydr. Polym..

[103] Yang J., Tian F., Wang Z., Wang Q., Zeng Y.-J., Chen S.-Q. (2008). Effect of chitosan molecular weight and deacetylation degree on hemostasis. J. Biomed. Mater. Res. Part B Appl. Biomater..

[104] Chou T.-C., Fu E., Wu C.-J., Yeh J.-H. (2003). Chitosan enhances platelet adhesion and aggregation. Biochem. Biophys. Res. Comm..

